# Homebrew: Protocol for glassmilk-based nucleic-acid extraction for SARS-CoV-2 diagnostics

**DOI:** 10.1016/j.xpro.2022.101300

**Published:** 2022-03-22

**Authors:** Robert Page, Edward Scourfield, Mattia Ficarelli, Stuart W. McKellar, Kwok Leung Lee, Thomas J.A. Maguire, Clement Bouton, Maria Jose Lista, Stuart J.D. Neil, Michael H. Malim, Mark Zuckerman, Hannah E. Mischo, Rocio T. Martinez-Nunez

**Affiliations:** 1 Department Infectious Diseases, School of Immunology and Microbial Sciences, Guy’s Campus, King’s College London, London SE1 9RT, UK; 2 South London Specialist Virology Centre, King’s College Hospital, London, UK; 3 ImmunoEngineering Lab, School of Cancer and Pharmaceutical Sciences, King's College London, Guy's Cancer Centre, Great Maze Pond, London SE1 9RT, UK

**Keywords:** Immunology, Microbiology, Molecular Biology

## Abstract

The gold standard protocol for severe acute respiratory syndrome coronavirus 2 (SARS-CoV-2) infection detection remains reverse transcription quantitative polymerase chain reaction (qRT-PCR), which detects viral RNA more sensitively than any other approach. Here, we present Homebrew, a low-cost protocol to extract RNA using widely available reagents. Homebrew is as sensitive as commercially available RNA extraction kits. Homebrew allows for sample pooling and can be adapted for automation in high-throughput settings.

For complete details on the use and execution of this protocol, please refer to Page et al. (2022).

## Highlights

• Homebrew is as sensitive as commercial RNA extraction kits for SARS-CoV-2 testing
• Homebrew only requires glassmilk, SDS, NaCl, isopropanol, ethanol, and water
• Cost- and time-effective compared with other RNA extraction techniques
• Homebrew allows for pooling of samples without loss of sensitivity

## Graphical abstract

**Figure:**
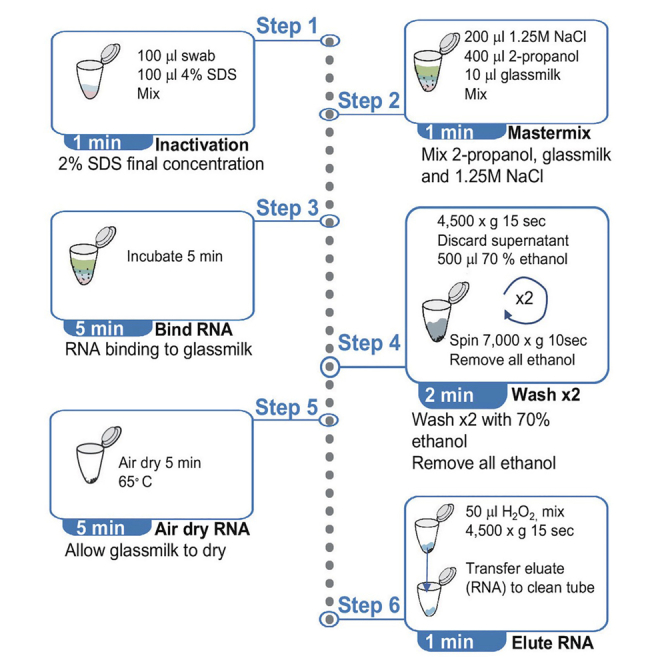

## Before you begin

***Note:*** This protocol allows for individual vs pooled samples. The first and second steps, inactivation and RNA binding, are done differently. Please choose one or another.

The protocol below describes the specific steps for using combined nose and throat swabs for SARS-CoV-2 detection employing the Centers for Diseases Control and Prevention (CDC) N1, N2 and RNaseP primers (Integrated DNA Technologies, 10006770) and TaqMan Fast Virus 1-Step Master Mix (Thermo Fisher, 4444434). All reagents below can be prepared in large batches, batch-tested and stored at ambient temperature (16°C–40°C). In our hands reagents did not lose activity over this temperature scale over more than 12 months storage.

We have made separate sections for testing individual and pooled samples. Details on the sensitivity of our pooling method can be found in Page et al. (2022).

### Institutional permissions

Samples for this study were provided under KCL TEST (KCL Ethics Ref: 21150); and as Service Delivery for King’s College Hospital. Samples were combined nose and throat swabs previously tested at KCL TEST or King’s College Hospital as part of a potential service development. All samples were anonymized and assessed after being diagnosed as SARS-CoV-2 positive by the hospital or negative by KCL TEST. We assessed a range of Ct values in order to represent a broad range of viral loads. Positive swabs were inactivated in a Category 3 facility employing 90°C 10 min in a dry bead bath as per Lista et al. (2021).

Abbreviations: GM: glassmilk; GM_MB: glassmilk master buffer.

### Preparation of the glassmilk

**Timing: 5 h**

This step describes how the matrix for nucleic acid capture is prepared.

Silicon dioxide (Sigma, 342890) once prepared will be referred as glassmilk, GM or silica matrix. The final concentration is 700 mg/mL and thus 21 g will render 30 mL of glassmilk, enough for 3,000 extractions.

1. Suspend 21 g silicon dioxide 325 mesh (Sigma 342890) in 40 mL 10% HCl in a 50 mL tube. Polypropylene tubes are appropriate.
  CAUTION: 10% HCl is highly corrosive. Only use under a fume hood when handling HCl. If diluting from a concentrated stock, add acid to water. This acid wash of silica matrix ensures getting rid of contaminants that may interfere with downstream qRT-PCR (e.g., RNAseP detection due to contamination).
  a. Agitate suspension for 4 h on a tube roller. Secure the cap with parafilm to minimize the risk of spillages. If no roller is available move every 10 min manually by gently inverting the tube 10 times.
  b. Centrifuge at 2,000 g for 5 min.
  c. Carefully remove HCl with a pipette and store in appropriate double container for reuse. HCl can be reused for this washing procedure multiple times. Do not dispose of HCl by pouring down the drain with copious amounts of water if it has not been previously neutralized. Spills may be neutralized with sodium bicarbonate or baking soda.

2. Resuspend the silica pellet with 40 mL single deionized water.
  a. Resuspend silica in a 50 mL polypropylene tube by agitation, vortexing at ∼ 134 × *g* or repeated tapping against a surface or hand so no HCl is ‘trapped’ in the pellet.
  b. Centrifuge at 2,000 g for 5 min.
  c. Remove wash water and dispose of through the drain. Flush with plenty of water.
3. Repeat step 2 for a total of 6 washes.
4. Before removing the final wash, measure pH of the supernatant with a strip, it should be between 7 and 8. If acidic pH is detected perform more water washes and measure pH after each wash until pH 7–8 is achieved.
5. Remove the supernatant, the pellet should now be white.
6. Resuspend silica in a final volume of 30 mL of H_2_O – if possible, use MilliQ or RNAse free H_2_O.
7. Aliquot in clean 1.5 mL tubes and keep at 16°C–40°C.

***Note:*** We have kept the glass milk for 18 months without loss in performance at temperature ranging from 4°C–40°C.

### Preparation of SDS, NaCl, and 70% ethanol

**Timing: 15 min**

8. Prepare 1.25 M NaCl.
  a. To prepare 500 mL 1.25 M NaCl, weight 36.525 g NaCl (Sigma/Merck S3014-500G) and dissolve in 500 mL H_2_O.
  b. Store at 16°C–40°C. 1.25 M NaCl is stable at 16°C–40°C for at least 6 months.
9. Prepare 70% EtOH.
  a. To prepare 500 mL, mix 350 mL pure EtOH (Fisher Scientific, 10644795) and 150 mL H_2_O.
  b. Ethanol should be kept in the dark if at all possible (by covering the tube with foil for example), at 16°C–40°C.
10. Prepare 4% SDS.
  a. From a 10% SDS stock, prepare 500 mL by mixing 200 mL 10% SDS (Fisher Scientific, 10552785) with 300 mL H_2_O.
  b. Keep SDS solution at 16°C–40°C.

***Note:*** We recommend purchasing a readily dissolved SDS stock.

***Note:*** Keep SDS solution at 16°C–40°C. SDS may precipitate at low temperatures; if precipitates are observed, please warm up and re-dissolve prior to use.

***Alternatives:*** SDS can also be prepared from powder. However, this is highly dangerous and needs to be performed under the fume-hood.

### Prepare glassmilk master buffer (GM-MB) for individual samples

**Timing: 5 min**

Prepare the GM_MB fresh every time prior to use. Follow this table for calculating volumes (in microliter) to prepare enough glassmilk master buffer GM_MB) for all samples. Consider always an excess of 10% (i.e., prepare enough for 11 samples if you are analyzing 10). The table below shows calculations for one and twenty-four samples.

**Table:** 

Samples #	10% added	Glassmilk (μL) Final 7 μg / sample	Isopropanol (μL) Final 65%	NaCl (μL) Final 0.4 M
1	N/A	10	400	200
10	11	110	4,400	2,200
24	26.4	264	10,560	5,280

**CRITICAL:** Make sure the master buffer is properly mixed and shake vigorously every 3 samples to avoid glassmilk pelleting.

### Prepare glassmilk master buffer (GM_MB) for pooled samples

**Timing: 5 min**

Prepare the GM_MB fresh every time prior to use.

Pooling samples can accelerate processing and considerably reduce the costs of sample processing. We have tested Homebrew and determined that the method allows for pooling of up to 19 negative samples with one positive sample (Page et al., 2022).

For use of the pooled protocol, we found that 20 μL glassmilk is sufficient to capture the nucleic acid material of up to 20 swabs and obtain a clearly visible pellet of glassmilk without losing sensitivity. If using less than 20 swabs, only adjust NaCl and isopropanol amounts. The glassmilk-master buffer recipe will change to:

**Table:** 

Samples #	10% added	Glassmilk (μL)	Isopropanol (μL)	NaCl (μL)
20	22	20	8,800	4,400
10	11	20	4,400	2,200
40	44	40	17,600	8,800

## Key resources table

**Table:** 

REAGENT or RESOURCE	SOURCE	IDENTIFIER
**Chemicals, peptides, and recombinant proteins**		

Silicon dioxide 325 mesh	Sigma/Merck	Cat# 342890
Carboxylate modified magnetic SpeedBeads^TM^	Sigma/Merck	Cat# GE45152105050250
SDS (500 g)	Sigma/Merck	Cat# L3771-500G
10% SDS (1 L)	Fisher Scientific	Cat# 10552785
NaCl (500 g)	Sigma/Merck	Cat# S3014-500G
NaI (sodium iodide)	Sigma/Merck	Cat# 383112-100G
GITC (Guanidine Isothiocyanate)	Sigma/Merck	Cat# 5120-250GM
Isopropanol (2.5 L)	Fisher Scientific	Cat# BP2618-212 2.5L
Absolute ethanol (500 mL)	Fisher Scientific	Cat# 10644795
Nuclease Free Water (500 mL)	Fisher Scientific	Cat# AM9930

**Critical commercial assays**		

TaqMan Fast Virus 1-Step Master Mix	Thermo Fisher Scientific	Cat# 4444434
2019-nCov CDC EUA Kit	Integrated DNA Technologies	Cat# 10006770

**Deposited data**		

Deposited in Mendeley Data: https://doi.org/10.17632/b2mscbnhmg.1		

**Experimental models: Organisms/strains**		

Severe acute respiratory syndrome-related coronavirus 2 (SARS-CoV-2)		Obtained from Public Health England

**Oligonucleotides**		

2019-nCoV_N1 Forward Primer: GAC CCC AAA ATC AGC GAA AT	CDC	2019-nCoV_N1-F 500 nM
2019-nCoV_N1 Reverse Primer: TCT GGT TAC TGC CAG TTG AAT CTG	CDC	2019-nCoV_N1-R 500 nM
2019-nCoV_N1 Probe: FAM-ACC CCG CAT TAC GTT TGG TGG ACC-BHQ1 NOTE: This probe spans the N:P13L mutation present in the Omicron variant (B.1.1.529)	CDC	2019-nCoV_N1-P 150 nM
2019-nCoV_N2 Forward Primer: TTA CAA ACA TTG GCC GCA AA	CDC	2019-nCoV_N2-F 500 nM
2019-nCoV_N2 Reverse Primer: GCG CGA CAT TCC GAA GAA	CDC	2019-nCoV_N2-R 500 nM
2019-nCoV_N2 Probe: FAM-ACA ATT TGC CCC CAG CGC TTC AG-BHQ1	CDC	2019-nCoV_N2-P 125 nM
RNAse P Forward Primer: AGA TTT GGA CCT GCG AGC G	CDC	RP-F 500 nM
RNAse P Reverse Primer: GAG CGG CTG TCT CCA CAA GT	CDC	RP-R 500 nM
RNAse P Probe: FAM – TTC TGA CCT GAA GGC TCT GCG CG – BHQ-1	CDC	RP-P 125 nM

**Software and algorithms**		

GraphPad Prism		https://www.graphpad.com/scientific-software/prism/
QuantStudio Software		QS5 (v.1.5.2) https://www.thermofisher.com/uk/en/home/global/forms/life-science/quantstudio-3-5-software.html QS7 Flex (v1.7.1): https://www.thermofisher.com/uk/en/home/global/forms/life-science/quantstudio-6-7-flex-software.html

## Materials and equipment

**Table:** 

Reagent	Final concentration	Amount
NaI (sodium iodide)	1.25 M	100 μL/100 μL swab
GITC (Guanidine Isothiocyanate)	1.25 M	100 μL/100 μL swab

Keep at 16°C–40°C.

**CRITICAL:** Both GITC and NaI are toxic. According to their MSDS: GITC has acute oral toxicity Category 4, acute dermal toxicity Category 4, acute Inhalation Toxicity - Dusts and Mists Category 4, skin Corrosion/Irritation Category 1 C and serious Eye Damage/Eye Irritation Category 1; NaI has acute oral toxicity Category 4, acute dermal toxicity Category 4, acute Inhalation Toxicity - Dusts and Mists Category 4, skin Corrosion/Irritation Category 1 C and serious Eye Damage/Eye Irritation Category 1.

***Alternatives:*** The homebrew method employs NaCl as its preferred chaotropic. NaCl is not considered hazardous by the 2012 OSHA Hazard Communication Standard (29 CFR 1910.1200). If for some reason NaCl was not available, NaI or GITC can be used instead as per Page et al., 2022.

## Step-by-step method details

***Note:*** When performing this protocol for the first time, we recommend either using spiked swab material with a known positive control (e.g., commercially available or laboratory-grown virus) or using swabs that have previously been tested by conventional extraction methods. Every extraction should include a positive control (in our case we employed laboratory-grown virus) and a negative extraction control (H_2_O). All steps in the extraction are performed at ambient temperature (16°C–40°C) unless otherwise stated.

We have performed this protocol on swabs from various different sources that have been stored at various temperatures (16°C to 40°C or −80°C) before analysis. As far as we can attest, all different viral transport media that are currently in circulation allow for successful isolation of viral material. Viral transport medium can be prepared as per CDC recommendation (https://www.cdc.gov/coronavirus/2019-ncov/downloads/Viral-Transport-Medium.pdf), i.e., 2% FBS 100 μg /mL Gentamicin 0.5 μg /mL Amphotericin B in Hanks Balanced Salt Solution (HBSS). Please refer to Page et al., 2022 for a more complete characterization of the robustness of this method.

### Inactivation and lysis of swab material (individual samples)

**Timing: 1 min**

This step inactivates potential SARS-CoV-2 viral particles and aids disruption of enveloped viral particles and subsequent solubilization of genetic material.

We recommend heat inactivation using a dry bead bath (either 70°C or 90°C for 10–30 min) prior to addition of SDS as it allows safe handling of otherwise infectious samples while preserving sensitivity of detection (Lista et al., 2021). Inactivated swabs must ALWAYS be opened in a microbiological safety cabinet class I when possible (class II when I not available) for the safety of the handler. Once swab material is non inactivated samples can be handled on a regular bench.

**CRITICAL:** If swab samples are not heat inactivated, add SDS in a microbiological safety cabinet.

1. Add 100 μL of 4% SDS per 100 μL of swab sample. Scale up appropriately if more swab sample is used. Incubation with SDS is not required.

**CRITICAL:** Check that there are no SDS precipitates. If precipitates are observed, place SDS in a lukewarm bath until precipitates are dissolved.

### Inactivation and lysis of swab material (pooled samples)

2. If using the pooled protocol, scale up the amount of 4% SDS accordingly. For example, for 20 samples of 100 μL each, add 20 × 100 μL (=2 mL) 4% SDS.

**CRITICAL:** If swab samples are not heat inactivated, add SDS in a microbiological safety cabinet.

**CRITICAL:** Check that there are no SDS precipitates. If precipitates are observed, place SDS in a lukewarm bath until precipitates are dissolved.

### Mastermix addition and RNA binding (individual samples)

**Timing: 6 min**

In this step, RNA from the lysed sample binds to glassmilk.

GM_MB contains NaCl as chaotropic agent, isopropanol to increase binding to glassmilk and glassmilk as RNA-binding matrix.

3. Prepare GM_MB buffer as per instructions above. Prepare enough for all samples and consider an extra 10% for pipetting errors.
4. Add 610 μL of GM_MB to 200 μL of lysed swab (100 μL 4% SDS + 100 μL swab sample).
5. Incubate 5 min at 16°C–40°C to allow RNA binding.

### Mastermix addition and RNA binding (pooled samples)

**Timing: 6 min**

6. Prepare GM_MB buffer as per instructions above. Prepare enough for all samples and consider an extra 10% for pipetting errors. This will contain 20 μL of glassmilk and a multiple per sample of 400 μL isopropanol and 200 μL 1.25 M NaCL. Due to the increased volume, pooled samples are mixed in a Falcon tube.
7. Mix well by vortexing.
8. Incubate 5 min at 16°C–40°C to allow RNA binding.

### RNA cleaning

**Timing: 3 min**

In this step, RNA from the lysed samples binds to glassmilk.

The bound RNA is pelleted and washed with 70% ethanol twice to remove excess proteins in the sample and increase the sensitivity of the PCR.

**Figure 1:**
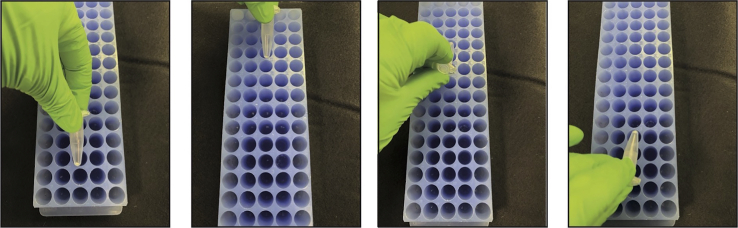
Resuspension of GM pellet by tapping against a tube rack
12. Add 500 μL of 70% ethanol and mix by flicking the tube.

***Note:*** Steps 11 and 12 can be inverted, i.e., add 500 μL of 70% ethanol to the GM pellet and then tap the plastic tube against a rack or vortexing to resuspend (Methods video S3, GM resuspension with ethanol).

**Figure 2:**
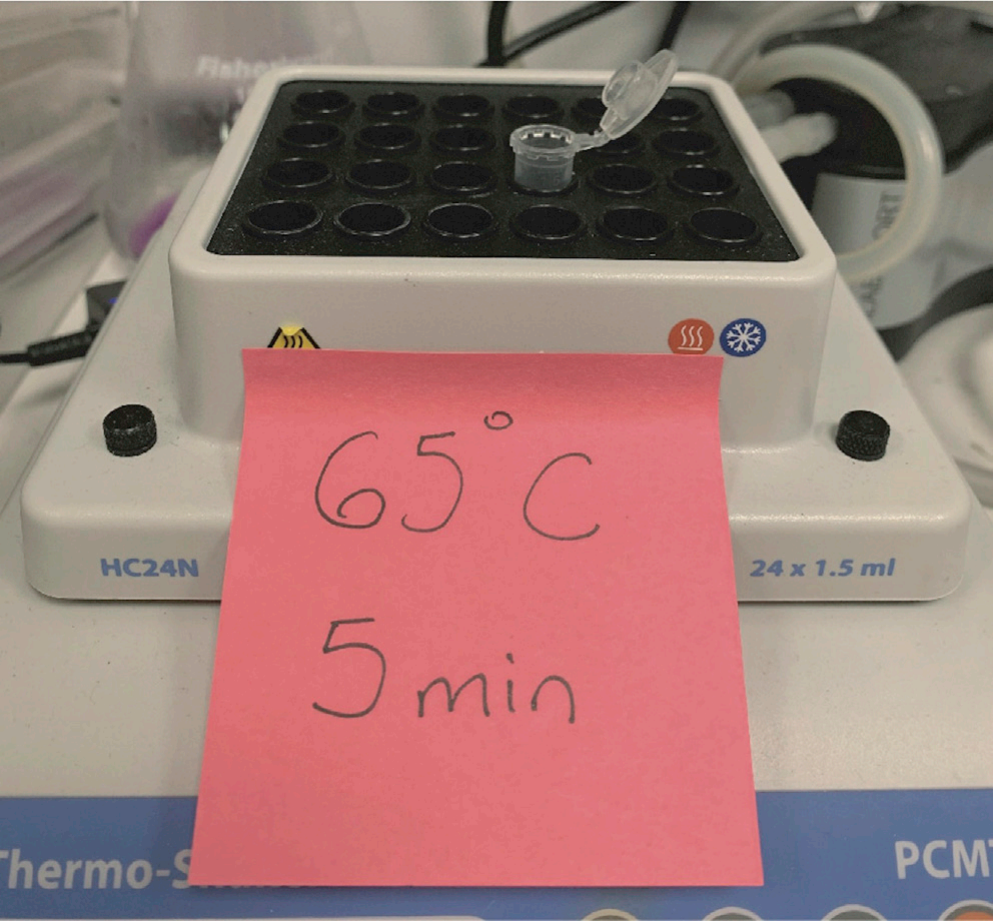
Example of air drying GM pellet in a heat block

9. Spin the sample at 4,500 g for 15 s.
10. Discard the supernatant by decanting into a waste basin. The pellet will not detach (Methods video S1: Decanting).
11. Disaggregate the pellet of GM by tapping the plastic tube against a rack or by vortexing (Figure 1 and Methods video S2, Disaggregation by tapping).
  a. If using the pooled protocol, resuspend the pellet from the Falcon tube and transfer with a pipette to a microcentrifuge tube. Continue processing as described for individual samples.

13. Spin the sample at 4,500 g for 15 s.
14. Discard the supernatant by decanting (Methods video S1, Decanting).
15. Repeat steps 11–14.
16. Spin the tube at 7,000 × *g* for 15 s to allow complete ethanol removal.
17. Remove all remaining 70% ethanol with a pipette.
18. Air dry at 65°C for 5 min in a heat block or equivalent to remove all ethanol from the pelleted GM with bound RNA (Figure 2).

Methods video S1. Decanting of supernatant This movie shows that the GM pellet remains attached to the tube, related to step 10.

Methods video S2. Disaggregation by tapping This movie shows resuspension of the GM pellet by tapping the plastic tube against a rack, related to step 11.

Methods video S3. GM resuspension with ethanol This movie shows addition of ethanol to the GM pellet prior to pellet resuspension by tapping the plastic tube against a rack, related to steps 10–12 and 12 note.

### RNA elution

**Timing: 1 min**

In this step RNA is released from the GM matrix in nuclease-free water.

To elute RNA from the dry GM matrix:

19. Resuspend pellet in 50 μL of nuclease-free water (Methods video S4, GM resuspension with water).
20. Spin the sample at 4,500 g for 15 s.
21. Take off 45 μL from the supernatant and transfer to a clean tube (Methods video S5, Elution).
22. Proceed to downstream detection of viral RNA. We routinely use qRT-PCR but other methods such as RT-LAMP (reverse transcription and loop-mediated isothermal amplification) or CRISPR-based methods can also be employed.

***Note:*** We employed 50 μL of nuclease-free water to resuspend the GM matrix. Higher volumes such as 70 μL may be employed but lower volumes will likely not resuspend the GM matrix and/or carry over matrix to the downstream reaction.

**Pause point:** Extracted RNA can be frozen at this step. Long term storage is recommended at −80°C but short-term storage (∼1 week) can be done at −20°C.

Methods video S4. GM resuspension with water This movie shows the resuspension of the dried GM pellet in 50 μl of water, related to step 19.

Methods video S5. Elution This movie shows the removal of 45 μl of supernatant containing RNA in a clean tube for downstream analysis, related to step 21.

### Reverse transcription and PCR

This step allows the detection of specific genes from SARS-CoV-2 in the eluted RNA, utilizing an internal human gene (RNAse P) as control of extraction.

Prepare the RT-PCR reaction master mix with the following format. Importantly, always include a non-template control, in which the RNA is replaced with H_2_O. When possible, prepare the PCR reaction and plate at a different location to that where swabs are handled to reduce risk of contamination. The positive control from the RNA extraction can be employed as positive control in the RT-PCR.

**Table:** qRT-PCR reaction master mix (TaqMan Fast Virus 1-Step Master Mix)

Reagent	Amount/reaction (μL)	10 reactions + 10% (μL) including 3 controls∗	24 reactions + 10% (μL) including 3 controls∗
RNA template	5		
Master Mix	5	55	132
Pre-mixed primer probe	1.5	16.5	39.6
ddH_2_O	8.5	93.5	224.4

**Table:** PCR cycling conditions (Fast mode)

Steps	Temperature	Time	Cycles
Reverse Transcription	50°C	5 min	1
Denaturation	95°C	20 s	1
Denaturation	95°C	3 s	45 cycles
Annealing/Extension	60°C	30 s

***Note:*** ∗ Controls must include positive and negative extraction controls and non-template PCR control.

***Note:*** For this step pooled and individual samples are treated equally. i.e., both are eluted in 50 μl water and 5 μl are used per technical replicate. For the purpose of establishing this protocol, we performed RT-PCR reactions in technical duplicates, for analytical purposes, it is custom to just perform one technical replicate. The limit of detection for our method, employing this master mix is Ct 36 (Page et al., 2022). Other qRT-PCR conditions may be employed (Lista et al., 2021; Reijns et al., 2020).

## Expected outcomes

Please refer to Page et al., 2022 for a complete description and troubleshooting of the method, including RNA binding capacity, limitation to isolate human cellular RNA or lack of effect of inhibitors such as blood in the quantification of SARS-CoV-2 in swab samples. Homebrew allows for effective isolation of RNA and not DNA. In the study by Page et al., we systematically compare Homebrew to QIAamp Viral RNA Mini Kit (QIAGEN) as per CDC-recommendation. We typically are within 1–3 Ct of the values obtained with RNA isolated using the QIAamp kit. Recovery of pure cellular total RNA is ∼30% from a 5 μg preparation (Page et al., 2022).

Although we recommend prior heat inactivation of swabs prior to the addition of SDS, SDS inactivates SARS-CoV-2 at concentrations as low as 0.5% (Patterson et al., 2020). We recommend prior heat inactivation to increase the speed of processing since all steps (including the addition of 4% SDS) can then be performed safely on a bench.

Quantification of SARS-CoV-2 material in RNA isolated from swabs using Homebrew has been validated employing the primer-probe and qPCR reagents listed above. According to our parameters, we consider negative/positive/inconclusive/void samples:

**Table:** Positive/negative/void/inconclusive thresholds

Result	Positive (Ct)	Negative (Ct)	Inconclusive (Ct)	Void (Ct)
N1	<36 regardless of N2 amplification	Undetermined	≥ 36 and N2 negative	
N2	<36 regardless of N1 amplification	Undetermined	≥ 36 and N1 negative	
RNAseP	< 35	< 35	< 35	> = 35

Inconclusive samples are those in which viral presence is difficult to determine, and void samples are those that have little presence of RNAseP, insufficient to determine a true negative result. In both cases, our recommendation is to ask for a new sample from the same individual, for testing as soon as possible. Void samples may be due to technical errors such as adding variable amounts of GM to each tube, which are reduced when preparing GM_MB rather than adding components separately. In our experience these are caused by insufficient biological material or wrong storage (e.g., repeated freeze-thaw cycles). We iterate the need for including a positive and a negative extraction control, as specified above. We recommend a positive control with a Ct of around 30 to minimize cross-contamination and demonstrate effective RNA extraction. Negative controls (extraction and qRT-PCR) should not amplify viral targets or RNAseP at all. Small contaminations with RNAseP may occur given its presence in plastics but should be kept at least at 5Ct difference from the maximum allowed.

As shown in the study by Page et al. (Page et al., 2022) we do not see a reduction in sensitivity when pooling up to 20 samples. Pooling is only recommended in low prevalence settings, since positive pools of samples must be reanalyzed to determine which sample(s) are positive among the pool, and it depends on the pool size (Cherif et al., 2020). In our case, positive rate should be less than 1 in 20 (5%), otherwise all pools of 20 samples will need to be re-tested given that each pool will (statistically) be positive

## Limitations

We have validated our method for SARS-CoV-2 RNA detection from combined nose and throat swab samples, including those that contain inhibitors such as blood (Page et al., 2022). Our method allows for the sub-optimal isolation of cellular RNA from cells and further modifications to the lysis step would be required to improve its efficiency and the quality of isolated RNA. We did not measure the variability between different batches of GM preparations. RNA extraction reproducibility was demonstrated employing serial dilutions of SARS-CoV-2 samples as well as comparable results to gold-standard clinically diagnosed swabs in ∼ 100 samples (Page et al., 2022). We recommend anyone starting to use Homebrew to carefully validate this protocol with swabs that have already been tested with alternative isolation methods until the user feels confident in our method. Although greatly decreasing the cost per sample, we have used our method with conventional qRT-PCR analysis. Sourcing the reagents for qPCR analysis is in our experience less of a bottleneck, but sufficient provision of reagents should be carefully planned in. Availability of real time thermocyclers is a limitation on the use of homebrew for qRT-PCR, but colorimetric and isothermal methods may still benefit from it (Alcántara et al., 2021; Baba et al., 2021; Joung et al., 2020).

Homebrew employing GM cannot be automated. However, our data employing carboxylated magnetic beads (Page et al., 2022) show comparable sensitivity, with those being amenable to automatization.

## Troubleshooting

### Problem 1

GM not resuspending after centrifugation.

### Potential solution

Pull tube over a microtube rack (Methods videos S1 and S2). This will create a vigorous small shaking that in our experience is most effective to resuspend the GM. Shake vigorously, vortex or flick the tube until resuspended.

### Problem 2

Positive extraction control failure.

**Figure 3:**
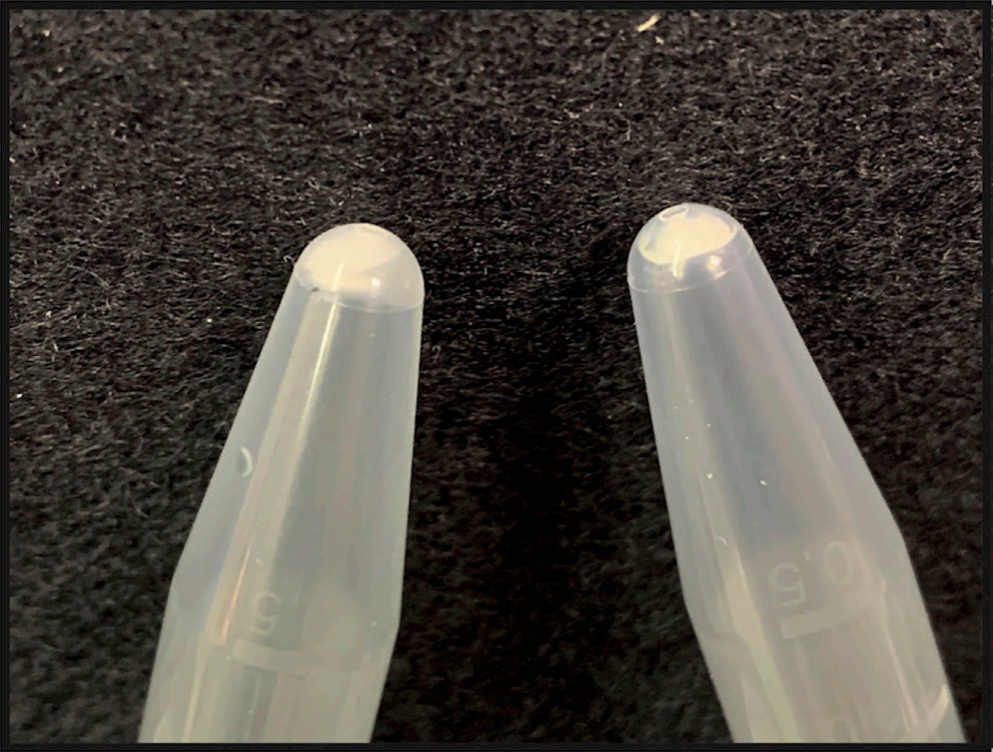
Wet versus dry GM pellet Left-over ethanol is inhibitory to PCR amplification (left). Completely dried pellet is white and defined (right).

GM contains ethanol in the last step. Carry over ethanol can negatively affect downstream processing i.e., transcription and/or PCR. A void result in the positive extraction control suggests that the extraction has not been efficient, and ethanol carry-over must be the first step to check. It is important to check if more than 50 μL is present in the tube after elution; typically GM absorbs 5 μL of the 50 μL and thus carry-over ethanol can be easily spotted at this stage. Figure 3 depicts a wet (left) *vs.* a dry (right) GM pellet.

### Potential solution

Air dry the pellet for 2 extra minutes.

### Problem 3

Multiple void results.

We typically consider a failed extraction batch when more than 10% of all samples extracted are ‘void’, i.e., there is no amplification of the internal control (RNAseP in our case). This is likely due to carry-over ethanol or deficient mixing of the GM-MB.

### Potential solution

Repeat the extraction of those samples (with positive and negative extraction controls) ensuring adequate mixing of GM-MB, incubation with the samples and air-drying of the GM pellet.

Ensure that all reagents have been filtered through a 0.2 μm PES membrane to remove contaminating RNases. Repeat extraction with a confirmed positive swab in parallel.

### Problem 4

Contamination of negative extraction control.

Given that PCR is an exponential reaction, it can be relatively easy to contaminate reagents with PCR products.

### Potential solution

To minimize this risk we recommend performing RNA extractions and qRT-PCR set up in different benches/rooms when possible. Aliquoting of negative and positive controls will also minimize risks of cross-contamination. Use of filter tips is highly recommended. If there is suspected contamination of one of the reagents discard and aliquot from the stock. If there is suspected contamination of the glassmilk, perform an acid wash as in the preparation steps.

### Problem 5

GM pellet is over dried.

We recommend air drying the GM pellet at 65°C for 5 min (Figure 2).

### Potential solution

If sample appears over dried i.e., the GM pellet does not resuspend (Methods video S4, GM resuspension with water), consider warming up water at 65°C prior to resuspending again. If this does not resuspend the GM pellet, we recommend re-extraction and careful monitoring of the drying process.

## Resource availability

### Lead contact

Further information and requests for resources and reagents should be directed to and will be fulfilled by the lead contact, Rocio T Martinez-Nunez (rocio.martinez_nunez@kcl.ac.uk).

### Materials availability

This study did not generate new unique reagents.

## Data Availability

Original data have been deposited to Mendeley Data: (https://doi.org/10.17632/b2mscbnhmg.1 from Page et al., 2022). Additional Supplemental Items are available from Mendeley Data at https://data.mendeley.com/datasets/t8wfrycwb5/1. According to UK research councils’ Common Principles on Data Policy, all data supporting this study will be openly available at https://doi.org/10.17632/b2mscbnhmg.1 from Page et al. (2022) and https://data.mendeley.com/datasets/t8wfrycwb5/1. According to Wellcome Trust’s Policy on data, software and materials management and sharing, all data supporting this study will be openly available at https://doi.org/10.17632/b2mscbnhmg.1 from Page et al. (2022) and https://data.mendeley.com/datasets/t8wfrycwb5/1.
